# Effects of Incubation of Human Brain Microvascular Endothelial Cells and Astrocytes with Pyridostigmine Bromide, DEET, or Permethrin in the Absence or Presence of Metal Salts

**DOI:** 10.3390/ijerph17228336

**Published:** 2020-11-11

**Authors:** Jessica F. Hoffman, John F. Kalinich

**Affiliations:** Internal Contamination and Metal Toxicity Program, Armed Forces Radiobiology Research Institute, Uniformed Services University, Bethesda, MD 20889, USA; jessica.hoffman@usuhs.edu

**Keywords:** Gulf War Illness, pyridostigmine bromide, permethrin, DEET, desert dust, metal, toxicity

## Abstract

Gulf War Illness (GWI) is a chronic, multi-symptom illness suffered by over one-third of American military veterans who served in the Persian Gulf War between 1990 and 1991. No current single-exposure scenario accounts for all the symptoms observed in GWI, and instead may be due to a multi-exposure scenario. As a larger effort to understand how one category of multi-exposure scenarios of organic compounds such as nerve gas prophylactic pyridostigmine bromide, or insecticides/pesticides such as N,N-diethyl-*m*-toluamide (DEET) and permethrin, plus heavy metals found in inhaled dust particles (Al, Fe, Ni, Sr, DU, Co, Cu, Mn, and Zn) might play a role in neural aspects of GWI, we begin this initial study to examine the toxicity and oxidative damage markers of human brain endothelial cell and human astrocyte cell cultures in response to these compounds. A battery of cytotoxicity assessments, including the MTT assay, Neutral Red uptake, and direct microscopic observation, was used to determine a non-toxic dose of the test compounds. After testing a wide range of doses of each compound, we chose a sub-toxic dose of 10 µM for the three organic compounds and 1 µM for the nine metals of interest for co-exposure experiments on cell cultures and examined an array of oxidative stress-response markers including nitric oxide production, formation of protein carbonyls, production of thiobarbituric acid-reactive substances, and expression of proteins involved in oxidative stress and cell damage. Many markers were not significantly altered, but we report a significant increase in nitric oxide after exposure to any of the three compounds in conjunction with depleted uranium.

## 1. Introduction

Gulf War Illness (GWI) refers to the chronic multi-symptom illness characterized by cognitive problems, fatigue, and muscle pain suffered by over one-third of American veterans who served in the Persian Gulf War in the period 1990–1991—close to 700,000 U.S. military personnel. A variety of exposures have been proposed as potential factors including nerve gas, vaccinations/prophylactics, depleted uranium, smoke from oil-well fires, stress, pesticides, and insecticides. However, the large number of personnel experiencing this multi-symptom illness did not all fit known single-exposure scenarios, suggesting instead a multi-exposure scenario. A review by [1] provides a good overview of the history and numerous studies around GWI.

Pyridostigmine bromide (PB), originally approved by the Food and Drug Administration (FDA) for treatment of myasthenia gravis, was given investigational status as a nerve gas prophylactic for the First Gulf War, but was not recommended for routine use. The requirement for informed consent and extensive record keeping was waived for the Department of Defense (DoD), however, and personnel were given blister packs of PB tablets and instructed to self-administer every 8 h [2,3]. Approximately a quarter-million personnel received at least one dose [4], but actual dose exposure to individuals is unknown. The U.S. Department of Agriculture (USDA) developed N,N-diethyl-*m*-toluamide (DEET) as an insecticide/insect repellent for the military in the 1940s, and it has had wide annual use [5]. DEET is rapidly absorbed by the skin and there are concerns that it could increase the uptake of other chemical, metal, or viral contaminants on the skin [6,7]. 3-(2,2-dichloroethenyl)-2,2-dimethylcyclopropanecarboxylic acid, known as permethrin (PM), is a synthetic pesticide used as an aerosol spray to treat the clothing and living spaces of military personnel during the Gulf War. Proper use is connected with no or minor adverse effects [8,9,10,11], but it can be toxic at high exposure levels, at least in animals [12,13].

Several studies have suggested that PB alone or in combination with other exposures, including stress, DEET, or PM, could be linked with illnesses in Gulf War veterans [14,15,16,17,18,19,20,21,22]. One study reported that treatment of mice with PB prior to virus exposure enabled the virus to cross the blood–brain barrier (BBB) and enter the brain, suggesting that although PB alone could not cross the BBB, it could increase the permeability of the barrier to other compounds [23]. Other studies report disruption of the BBB and significant damage in specific areas of the brain [24,25,26] as well as neurological deficits [27,28,29,30].

Other studies suggest that respiratory exposure to the fine-grained sand particles found in the area, deemed “desert dust,” could also be linked to GWI [31,32]. Analysis of this desert dust found high levels of a variety of metals, including aluminum, iron, uranium, nickel, cobalt, copper, lead, chromium, strontium, tin, manganese, zinc, barium, arsenic, and vanadium, as well as microbial contaminants [33,34,35]. The U.S. Army Center for Health Promotion and Preventive Medicine (now the U.S. Army Public Health Command) estimates that during Operation Iraqi Freedom and Operation Enduring Freedom, particulate levels exceeded the Military Exposure Guidelines (MEG) over 97% of the time [36]. There is growing evidence that metals internalized through ingestion, inhalation, or wounds, cross the BBB and induce neuronal injury, behavioral changes, and development of neurological diseases [37,38,39,40,41,42,43,44,45,46]. Once accumulated in the brain, many metals induce oxidative stress and alter essential trace metal pools in various regions of the brain, further affecting neurological function [47,48,49].

Our overall hypothesis is that combined exposures of PB, PM, and DEET adversely affect BBB permeability, allowing metals solubilized from inhaled desert dust particles to enter the brain and result in extensive oxidative damage. In order to begin testing this hypothesis and establish an in vitro BBB model system, we first assessed viability and function of human neural cells after individual or concurrent exposure to PB, PM, DEET, and multiple metals identified in desert dust using in vitro cell cultures of human brain microvascular endothelial cells (BMECs) and human astrocytes and report those results herein.

## 2. Materials and Methods

### 2.1. Cells and Cell Culture

#### 2.1.1. Human Astrocytes

Normal human astrocytes (male, CC-2565, Lonza, Walkersville, MD, USA) were used between passages 2 and 6 for the initial toxicity studies. However, because of vendor delays, further studies were completed using normal human astrocytes (male, #1800) from ScienCell Research Labs (Carlsbad, CA, USA). Cells were maintained in Dulbecco’s Minimal Essential Medium (D-MEM) with Glutamax (Gibco, Grand Island, NY, USA) supplemented with N2 Supplement CTS (Gibco) and Fetal Bovine Serum One-Shot (10%, Gibco) and were not used past passage 8 for all other experiments. 

#### 2.1.2. Human Brain Microvascular Endothelial Cells

Human brain microvascular endothelial cells (male, ACBRI 376) were obtained from Cell Systems (Kirkland, WA, USA) and cultured in Vasculife VEGF Basal Medium supplemented with the associated VEGF LifeFactors Kit (Lifeline Cell Technology, Frederick, MD, USA) with the following modifications. The LifeFactors Kit fetal bovine serum was not used and only 10 mL of the LifeFactors-provided L-glutamine was used as a supplement. In addition, iCell Endothelial Cell Medium Supplement (Cellular Dynamics International, Madison, WI, USA) was added to 10%. Cells were used between passages 3 and 6 for the initial toxicity studies and not past passage 8 for all other experiments. 

#### 2.1.3. Culture Setup

Stock cell cultures were maintained in tissue culture flasks (75 cm^2^ area) at 37 °C in a humidified atmosphere of 5% CO_2_. For endothelial cells, the flasks were treated with 6 mL of Quick-Coat (Angio-Proteomie, Boston, MA, USA) to provide a suitable substrate for attachment. For both cell lines, medium was refreshed every other day and cells were subcultured when 70–80% confluent based upon direct microscopic observation. Briefly, medium was aspirated from the flasks and the flasks were washed once with phosphate-buffered saline (PBS, Gibco). After aspirating the PBS, 3 mL of StemPro Accutase Cell Dissociation Reagent (Gibco) were added and the flask left at room temperature for up to 8 min to allow the cells to dissociate from the flask. Once detached, fresh medium was added, and the cell suspension pipetted to break up any clumps. The cell suspension was centrifuged at 240× *g* for 10 min, after which the cell pellet was resuspended in a suitable amount of the appropriate medium and transferred to new flasks. Cell numbers and viability were determined using trypan blue (Gibco) exclusion with the Bio-Rad Model TC-20 Cell Counter (Hercules, CA, USA). 

#### 2.1.4. Fluorescent Immunohistochemistry of BMEC Markers

BMEC cells were cultured onto 8-chamber slides (Nunc LabTek II, Fisher Scientific, Pittsburgh, PA, USA) as described in Section 2.3.1, then fixed with 100% methanol chilled at −20 °C for 5 min and washed 3 times with ice cold PBS. Chambers designated for ZO-1 staining were permeabilized by incubation with 0.1% Triton X-100 in PBS for 10 min, then washed 3 times in 5 min washes of PBS. Chambers designated for occludin staining were not permeabilized. All chambers were then incubated with 1% BSA, 22.52 mg/mL glycine in PBST (PBS + 0.1% Tween20) for 30 min to block non-specific binding. Chambers were then incubated with 1% BSA PBST (without glycine) and the designated dilution of primary antibody ZO-1 or occludin overnight at 4 °C. Chambers were then decanted, washed 3× in PBS 5 min each, and incubated with appropriate secondary antibody in 1% BSA PBS for 1 h at room temp in the dark, followed by a decant and 3 washes of PBS, 5 min each. Chambers were then counterstained with 1 µg/mL DAPI for 1 min and rinsed with PBS. Chamber sides were removed, coverslipped with permount, and imaged as described in Section 2.3.1. Antibody information is presented in Supplementary Materials Table S1, and images of overlaid target antibody with DAPI are presented in Supplementary Materials Figure S1.

### 2.2. Organics and Metals for Treatment

#### 2.2.1. Organic Compounds of Interest

The following compounds were tested, pyridostigmine bromide (PB), permethrin (PM), and N,N-diethyl-*m*-toluamide (DEET) (all from Sigma Chemical, St. Louis, MO, USA). Stock solutions were prepared in dimethylsulfoxide (DMSO, Invitrogen, Carlsbad, CA, USA) before dilution to appropriate working concentrations in cell culture medium.

#### 2.2.2. Metals of Interest

Metals to be tested included aluminum (Al as aluminum chloride hydrate), iron (Fe as iron chloride hexahydrate), uranium (depleted uranium as uranyl nitrate hexahydrate), nickel (Ni as nickel chloride hexahydrate), cobalt (Co as cobalt chloride hexahydrate), copper (Cu as copper chloride dihydrate), strontium (Sr as strontium chloride hexahydrate), manganese (Mn as manganese chloride tetrahydrate), and zinc (Zn as zinc chloride). All metals were obtained from Sigma Chemical except for uranyl nitrate hexahydrate which was from Fluka Chemical (Ronkonkoma, NY, USA). Metals were dissolved in the appropriate cell culture medium and diluted to provide proper working concentrations. 

### 2.3. Viability and Toxicity Assays to Determine Sub-toxic Exposure Dose

#### 2.3.1. Cell Culture

Cells were plated onto 96-well plates at 5000 cells/well (astrocytes) or 2500 cells/well (BMEC) 100 μL per well, and allowed to incubate at 37 °C, 5% CO_2_ for 24 h. Media was then removed and cells treated with media spiked with metal or organic compounds in a serial dose dilution ranging from 0 μM to 1000 μM and incubated for an additional 24 h prior to viability assays. For images, cells were plated at 50,000 cells/chamber on Nunc Lab-Tek II 8-chamber slides, treated in the same manner as the plates. Medium was aspirated, the chambers washed with Dulbecco’s PBS (DPBS, Gibco) and cells fixed with ice-cold methanol for 5 min. After removing the methanol, the slides were air dried before being stained with Giemsa (Gibco) stain (1:20 dilution of stock) for 10 min followed by extensive washing with tap water. Images were taken with an Olympus BX61 Microscope with D72 camera and cellSens Entry Software (version 1.5) (Olympus America, Melville, NY, USA). 

#### 2.3.2. MTT Assay

Metabolic viability was assessed using the CellTiter 96^®^ Aqueous One Solution Cell Proliferation Assay kit (Promega Corporation, Madison, WI, USA). The assay is based upon the ability of dehydrogenase enzyme systems, located in the cell mitochondria, to reduce a tetrazolium compound to a colored formazan product, which is easily detected colorimetrically. Briefly, after the 24 h treatment incubation period, 20 µL of CellTiter 96^®^ Aqueous One Solution Reagent was added to each plate well, incubated 1 h, then absorbance determined at 490 nm using a microplate reader (SpectraMax Model 250 Microplate Spectrophotometer, Molecular Devices Corporation, Sunnyvale, CA, USA). Metabolic viability of the treated cells was normalized to the media-only control cells.

#### 2.3.3. Neutral Red (NR)

Cell toxicity was assessed using the In Vitro Toxicology Assay Kit (Sigma Chemical). Neutral Red dye (Toluylene Red) is only taken up by viable cells via active transport, thus a decrease in the amount of dye released from cell culture indicates more cytotoxic conditions. Briefly, after the 24 h treatment incubation period, 10 μL Neutral Red dye was added to each plate well, incubated for 2 h, washed twice with 100 μL DPBS, and incubated at room temperature for 10 min with 100 μL Stabilizing Solution in each well for 10 min prior to determining absorbance at 540 nm using a microplate reader. Viability of the treated cells was normalized to the media-only control cells.

### 2.4. BMEC Culture Experiments for Protein Expression and Oxidative Damage Assessments

The following procedure was used to provide samples to determine protein expression and assess oxidative damage of endothelial cells treated with metals in the presence or absence or Gulf War-associated organic compounds. The basic experimental plan was to plate the cells on Day 1, replace the medium on Day 2, add organic compounds (if required) on Day 3, add metals (if required) on Day 4, and harvest on Day 5. Briefly, BMECs were harvested as above and re-plated on 6-well tissue culture plates at a concentration of 2 × 10^5^ cells/well and the plates returned to the incubator. The next day the medium was aspirated and replaced with fresh medium (2 mL/well). If required, on the next day the appropriate organic compound was added to a non-toxic dose (as determined above) and the plates returned to the incubator. The next day, if required, appropriate metals were added, again at a non-toxic dose as previously determined and the plates returned to the incubator. The next day, 1 mL of medium was removed from each well, placed in a 1.5 mL tube and centrifuged at 18,400× *g* for 10 min to pellet any detached cells. The supernatant (0.8 mL) was then removed, placed in a fresh 1.5 mL tube and stored at −80 °C until needed for the nitric oxide assay. The remaining medium in the wells was aspirated and the wells washed with 2 mL of PBS (Gibco). After aspirating the PBS, 1 mL of Accutase was added to each well and the plates incubated at room temperature for 8 min to detach the cells. The cell suspension was placed in a 1.5 mL tube and centrifuged at 18,400× *g* for 10 min to pellet the cells. After removing the supernatant, the cell pellets were stored at −80 °C until required for protein expression analysis and oxidative damage assessments. 

### 2.5. Reactive Nitrogen Species

The Nitrate/Nitrite Colorimetric Assay Kit (#78001, Cayman Chemical, Ann Arbor, MI, USA) was used analyze nitric oxide (NO) levels in the collected cell culture supernatants. The final products of nitric oxide are nitrate and nitrite, but since the relative proportions of these compounds can be variable, the best assessment of total nitric oxide production is the sum of both of these. This is accomplished by converting the nitrate in the sample to nitrite using nitrate reductase, then converting the nitrite to an azo compound using the Griess reaction. Briefly, 80 µL of cell supernatant was mixed with 10 µL of enzyme cofactor mixture and 10 µL of nitrate reductase mixture in a 96-well plate. The plate was incubated at room temperature for 1 h, after which the Griess reagents provided in the kit were added to each well. After 10 min at room temperature, absorbance was read at 540 nm in a BioTek Synergy Model H1M Multimodal Plate Reader with GEN5 Software (BioTek Instruments, Winooski, VT, USA) and the amount of nitrate determined from a standard curve and expressed in µM. 

### 2.6. Lipid Peroxidation (TBARS) Assay 

The effect of the various treatments on the induction of lipid peroxidation was determined using the thiobarbituric acid-reactive substances (TBARS) assay from Cayman Chemical (kit #10009055). The stored cell pellets collected from the 6-well plates were resuspended in 200 µL of PBS and homogenized with a sonicator (Fisherbrand Model 120 Sonic Dismembranator, Pittsburgh, PA, USA) for 5 s with a 1 s pulse at 40% amplitude. Total protein from each sample supernatant was measured by Bio-Rad Protein Assay (Bio-Rad Laboratories, Hercules, CA, USA, cat #500-0006), in duplicate, against a BSA standard curve, on a spectrophotometer (BioTek Synergy Model H1M Multimodal Plate Reader with GEN5 Software), and read at 595 nm. Briefly, 100 µL of cell homogenate was mixed with 100 µL of TCA Assay Reagent and 800 µL of Color Reagent (both provided in the kit) and the tubes placed in a heat block at 95 °C for 60 min. They were then placed on ice for 10 min before centrifugation for 10 min at 1600× *g*. Supernatant (2 × 100 µL aliquots) were removed and placed in a black plate for fluorescence measurement using 530 nm excitation and 550 nm emission in a BioTek Synergy Model H1M Multimodal Plate Reader with GEN5 Software. The amount of thiobarbituric acid-reactive substance in the samples was quantitated using a standard curve prepared with the kit-provided TBA Malondialdehyde Standard with results normalized to protein content. 

### 2.7. Protein Carbonyl Levels 

Oxidative damage to protein side chains was assessed using the protein carbonyl assay. The Abcam Protein Carbonyl Content Fluorometric Assay Kit (ab235631, Abcam, Cambridge, MA, USA) was used for this purpose. Cell homogenates (50 µL), prepared as above, were added to 50 µL FTC fluorophore dissolved in Protein Carbonyl Assay Buffer (both provided in the kit) in a 1.5 mL tube. The tube was mixed and allowed to sit overnight at room temperature in the dark. Next, 200 µL of ice-cold 20% trichloroacetic acid was added and the tubes left on ice for 10 min. After this the tubes were centrifuged at 10,000× *g* for 10 min, the supernatant removed, and the resulting pellet washed 3× with 200 µL of 100% isopropanol. After the last wash step, the tubes were allowed to dry at room temperature for 1 h. The pellet was resuspended in 50 µL of 6 M guanidine and heated at 50 °C for 2 h. After cooling to room temperature, 450 µL of Sample Dilution Buffer from the kit was added. Two 100 µL aliquots were taken and placed in a black 96-well plate for fluorescence measurement at 485 nm excitation and 535 nm emission in a BioTek Synergy Model H1M Multimodal Plate Reader with GEN5 Software. The amount of protein carbonyls in the samples was determined by comparing to a standard curve prepared from the FTC fluorophore. In addition, the amount of protein contained in the processed sample, after addition of the Sample Dilution Buffer, was determined using bicinchoninic acid (BCA Protein Assay Kit (#23277), Pierce Biotechnology, Rockford, IL, USA). Protein carbonyl values were normalized to protein content. 

### 2.8. Protein expression using the ProteinSimple Wes

Cell pellets were re-suspended in RIPA buffer (Thermo, Waltham, MA, USA, cat#89901) plus Halt Protease and Phosphatase Inhibitor Cocktail (Thermo cat#78442) and homogenized in a Bullet Blender (Next Advance, Troy, NY, USA) with 0.5 mm glass beads (NextAdvance, cat#GB05-RNA) (settings speed 6, 5 min × 2 runs) and then centrifuged at 1340× *g* for 10 min. Total protein from each sample supernatant was measured by Bio-Rad Protein Assay (Bio-Rad Laboratories, cat #500-0006), in triplicate, against a BSA standard curve, on a spectrophotometer (BioTek Synergy Model H1M Multimodal Plate Reader with GEN5 Software), and read at 595 nm. Proteins of interest were quantified using an automated capillary-based size sorting chemiluminescent system ‘WES’ from ProteinSimple (San Jose, CA, USA). All procedures were performed with manufacturer’s reagents (12-235 kDa kit, cat#SM-W004-1 and PS-ST01EZ-8) according to the user manual with some adjustments: samples are aliquoted to 1 μg/μL before mixing 4 μL with 1 μL fluorescent master mix, then denatured at 94 °F (34.4 °C) for 4 min, given a quick spin, and loaded on the plate at 4 μL per well. In run settings, stacking time was changed to 18 s, separation time to 31 min, and antibody diluent time to 30 min. Antibody information is listed in Supplementary Materials Table S1. Peak values were determined using Compass Software (ProteinSimple). Target proteins are presented as a ratio of the target protein expression normalized to within-sample β-actin expression in arbitrary units. Since the ProteinSimple Wes system uses capillary size separation and chemiluminescent detection, an example of the raw data run for one of the GPX4 DEET + metal experiment sample sets is shown in Supplementary Materials Figure S6a, and a traditional Western blot image derived from that data is in Supplementary Materials Figure S6b.

### 2.9. Statistical Analyses

Data in the figures represent the mean and standard deviation of three independent experiments. Analyses were performed using GraphPad Prism Software (version 8.4.2, La Jolla, CA, USA). One-way ANOVAs with Sidak’s multiple comparisons post-hoc test were performed, with specific group comparisons or any other statistical analyses noted. Specific post-hoc *p* values less than 0.05 were considered significant.

## 3. Results

We hypothesize that exposures of sub-toxic levels of DEET, PB, and PM, particularly in combination with concurrent exposure to sub-toxic levels of metals, adversely affect the health and viability of cells involved in the blood–brain barrier (BBB), which could lead to subtle changes in permeability and function. To test this, human brain endothelial cells and human astrocytes exposed to DEET, PB, and PM, nickel, cobalt, strontium, zinc, manganese, copper, iron, aluminum, or depleted uranium, individually or in combination, were assessed for toxicity and viability using standard cell culture techniques.

### 3.1. Determination of Sub-toxic Doses of Organic and Metal Compounds

We began by identifying a sub-toxic dose of each compound to be used in experiments to examine subtle changes in blood–brain barrier integrity rather than outright or extensive damage. Monocultures of BMECs or astrocytes were exposed to each organic or metal compound of interest in a dose range of 1–1000 μM and assessed for toxicity using MTT and NR assays. MTT measures metabolic activity, and measurements after treatment were normalized back to media-only controls and presented as percent of control, where 100% represents no change from control (0 μM compound) and indicated as a horizontal dashed line in graphs. Values below this line indicate reduced metabolic activity, and thus are considered toxic. Similarly, the same experimental design was conducted and assayed with Neutral Red, which measures cell viability. Measurements are also presented as percent of control, and values below this line indicate fewer viable cells, and thus are toxic doses. MTT and NR results for BMECs and astrocytes exposed to organic compounds are presented in Supplementary Materials Figure S2, MTT and NR results for BMECs exposed to metals are presented in Supplementary Materials Figure S3, and MTT and NR results for astrocytes exposed to metals are presented in Supplementary Materials Figure S4. Additionally, cells were also cultured on slides, exposed to the same compound dose ranges, and stained with Giemsa to visualize changes in cell structure (BMECs images are presented in Supplementary Materials Figure S5a,b, astrocyte images are presented in Supplementary Materials Figure S6a,b). No quantitative analyses were conducted on the images. As expected, increasing doses of organic compounds and metals were increasingly toxic to both endothelial and astrocyte cell cultures. For most organic compounds, the 10 μM concentration is at or near the line of no change from control while the 100 μM dose is a subtle to sharp drop below normal. There is more variation in toxicity patterns with the metals, but for most metals, the 1 μM concentration is at or near the line of no change from control while the 10 μM dose is a decrease from control for several metals, and 100 μM is a drastic decrease in viability for most metals. We decided that 10 μM would serve as the sub-toxic dose for organic compounds and 1 μM would serve as the sub-toxic dose for metals for experiments moving forward.

**Figure 1:**
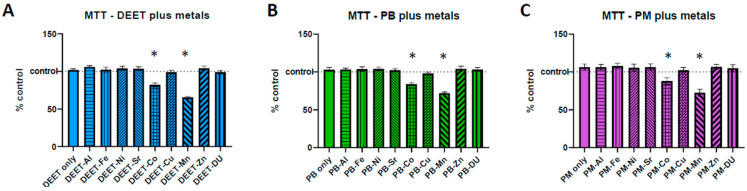
Toxicity of organic and metal combination exposures on brain microvascular endothelial cells (BMEC). Toxicity assessment via MTT assay on BMEC cultures for combinations of an each organic compound with individual metals. (**A**) DEET (10 µM) plus metals (1 µM), (**B**) pyridostigmine bromide (PB) (10 µM) plus metals (1 µM), and (**C**) permethrin (PM) (10 µM) plus metals (1 µM). Dotted line at 100 indicates 100% of control measurement: values below the line indicate that the dose is toxic to the cells and viability is decreased. Data represent the mean and standard deviation of three independent experiments. An * denotes a statistically significant difference from control at *p* < 0.05.

To determine whether there was any synergistic toxicity effects of co-exposures of organics and metals to BMECs, monocultures of BMECs were exposed to a media only control, 10 µM of an organic alone, or the organic in combination with one of the metals at 1 µM and assayed and analyzed via MTT test as above, where 100% represents no change from the media-only control. One-way ANOVAs were significant for all three experimental sets in Figure 1A, DEET + metals F_(9,20)_ = 29.28, *p* < 0.0001; Figure 1B, PB + metals F_(9,20)_ = 21.17, *p* < 0.0001; Figure 1C, PM + metals F_(9,20)_ = 7.80, *p* < 0.0001). None of the organic compounds alone were significantly different from control (one-sample *t*-test compared to 100, DEET *t* = 1.32, df = 2, *p* = 0.32; PB *t* = 1.10, df = 2, *p* = 0.39; PM *t* = 1.58, df = 2, *p* = 0.26). However, Co and Mn in conjunction with all three organic compounds were significantly more toxic to BMECs than the corresponding organic compound alone (Sidak post-hoc: DEET vs. DEET + Co * *p* < 0.0001; DEET vs. DEET + Mn * *p* < 0.0001; PB vs. PB + Co * *p* = 0.0001; PB vs. PB + Mn * *p* < 0.0001; PM vs. PM + Co * *p* = 0.0310; PM vs. PM + Mn * *p* < 0.0001).

### 3.2. Nitric Oxide (NO) Production

**Figure 2:**
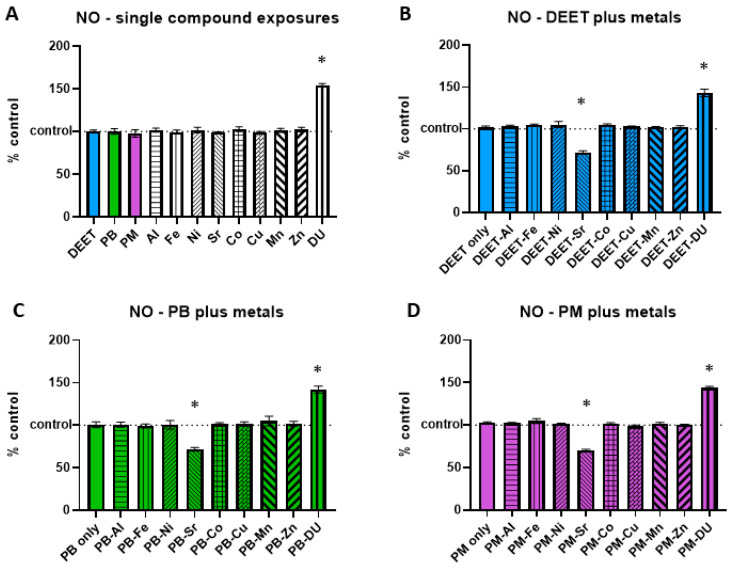
Nitric oxide (NO) in brain microvascular endothelial cell (BMEC) cultures after organic compound and metal exposure. Assessment of nitric oxide (NO) levels in BMEC cultures after exposure to organic compounds and/or metals. (**A**) organic compounds (10 µM) or metals (1 µM) alone, (**B**) DEET (10 µM) plus metals (1 µM), and (**C**) pyridostigmine bromide (PB) (10 µM) plus metals (1 µM) and (**D**) permethrin (PM) (10 µM) plus metals (1 µM). Dotted line at 100 indicates 100% of control measurement; values above the line indicate an increase in NO in the culture. Data represent the mean and standard deviation of three independent experiments. An * denotes a statistically significant difference from control at *p* < 0.05.

NO increases in response to cell stress or damage. BMEC cell cultures were exposed to a media-only control, organic compounds and metals singly, or in sets of each organic alone and in combination with each metal. Within each experiment, NO signal was measured and normalized to the media-only control value and presented as percent of control, were 100% represents no change from control and indicated as a horizontal dashed line in graphs. Values above this line indicate an increase in NO concentration, and thus a status of more stressed or damaged cell culture. For each of the single exposure conditions (Figure 2A), a one-sample *t*-test of each % of control value is compared back to a value of 100 to determine if the treatment is different from the media-only control it was normalized to. Of all the organic compounds and metals alone, only DU was significantly different (*t* = 30.80, df = 2, * *p* = 0.0011), increasing NO levels over media-only control cells.

In the DEET or DEET + metals exposure experiment set (Figure 2B), DEET alone was not significantly different compared to media control (a one-sample *t*-test vs. 100%, *t* = 0.97, df = 3, *p* = 0.40). A one-way ANOVA (F_(9,30)_ = 58.55, *p* < 0.0001) with Sidak’s multiple comparisons test compared each DEET + metal exposure back to DEET alone. Similar to the effect of DU alone, DEET + DU also had a significantly higher production of NO compared to DEET alone (* *p* < 0.0001). DEET + Sr was significantly lower in NO production than DEET alone (* *p* < 0.0001), indicating a possible protective effect of Sr. 

The PB or PB + metals exposure experiment set (Figure 2C, F_(9,30)_ = 23.20, *p* < 0.0001) had similar effects to that of the DEET + metal set. PB alone was not significantly different from the media control (*t* = 0.15, df = 3, *p* = 0.89), but PB + DU had a significantly higher production of NO compared to PB alone (* *p* < 0.0001) and PB + Sr appears to have a protective effect compared to PB alone (* *p* < 0.0001).

The PM or PM + metals exposure experiment set (Figure 2D, F_(9,30)_ = 137.1, *p* < 0.0001) also had similar effects to that of the other two organic + metal sets. PM alone was not significantly different from the media control (*t* = 2.48, df = 3, *p* = 0.09), but PB + DU had a significantly higher production of NO compared to PB alone (* *p* < 0.0001) and PB + Sr appears to have a protective effect compared to PB alone (* *p* < 0.0001).

### 3.3. Thiobarbituric Acid-Reactive Substances (TBARS)

**Figure 3:**
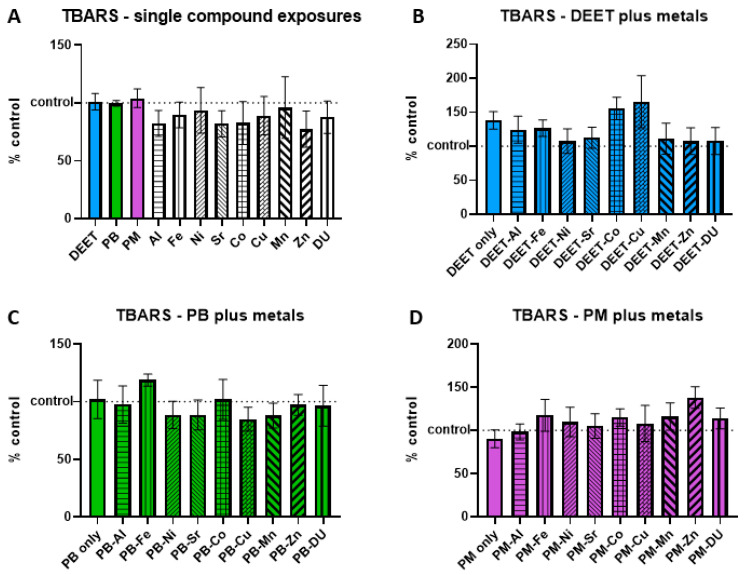
Thiobarbituric acid-reactive substances (TBARS) in brain microvascular endothelial cell (BMEC) cultures after organic compound and metal exposure. Assessment of thiobarbituric acid-reactive substances (TBARS) levels in BMEC cultures after exposure to organic compounds and/or metals. (**A**) organic compounds (10 µM) or metals alone (1 µM), (**B**) DEET (10 µM) plus metals (1 µM), (**C**) pyridostigmine bromide (PB) (10 µM) plus metals (1 µM), and (**D**) permethrin (PM) (10 µM) plus metals (1 µM). Dotted line at 100 indicates 100% of control measurement; values above the line indicate an increase in TBARS in the culture. Data represent the mean and standard deviation of three independent experiments.

Thiobarbituric acid-reactive substances (TBARS) are formed as a byproduct of lipid peroxidation, and an increase is indicative of cell oxidative stress/damage. BMEC cell cultures were exposed to a media-only control, organic compounds and metals singly, or in sets of each organic alone and in combination with each metal. Within each experiment, TBARS signal was measured and normalized to the media-only control value and presented as percent of control, where 100% represents no change from control and indicated as a horizontal dashed line in graphs. Values above this line indicate an increase in TBARS concentration, and thus a status of more stressed or damaged cell culture. In the single-compound experiments, no metal or organic compound was significantly different in a one-sample *t*-test of each % of control value is compared back to a value of 100 (Figure 3A). Similarly, there were no significant differences in any of the organic + metal compound groups, either between the organic compound alone compared to control, or any of the organic compound + metal groups compared back to the corresponding compound only exposure (Figure 3B) DEET + metals, ANOVA F_(9,30)_ = 1.03, *p* = 0.44; (Figure 3C) PB + metals, ANOVA F_(9,30)_ = 0.55, *p* = 0.82; (Figure 3D) PM + metals, ANOVA F_(9,30)_ = 0.76, *p* = 0.65).

### 3.4. Protein Carbonyl

**Figure 4:**
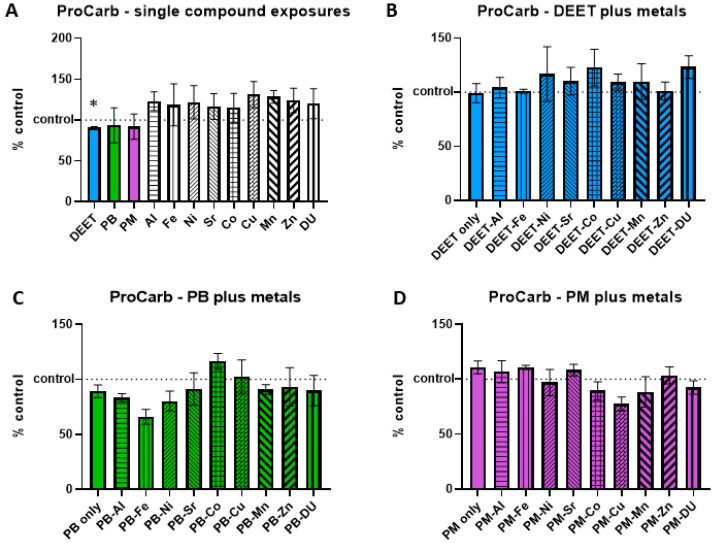
Protein carbonyl (ProCarb) in brain microvascular endothelial cell (BMEC) cultures after organic compound and metal exposure. Assessment of protein carbonyl levels in BMEC cultures after exposure to organic compounds and/or metals. (**A**) organic compounds (10 µM) or metals alone (1 µM), (**B**) DEET (10 µM) plus metals (1 µM), (**C**) pyridostigmine bromide (PB) (10 µM) plus metals (1 µM), and (**D**) permethrin (PM) (10 µM) plus metals (1 µM). Dotted line at 100 indicates 100% of control measurement; values above the line indicate an increase in protein carbonyl in the culture. Data represent the mean and standard deviation of three independent experiments. An * denotes a statistically significant difference from control at *p* < 0.05.

Protein carbonyl groups are carbonyl groups that form on protein side chains upon oxidation. BMEC cell cultures were exposed to a media-only control, organic compounds and metals singly, or in sets of each organic alone and in combination with each metal. Within each experiment, protein carbonyl signal was measured and normalized to the media-only control value and presented as percent of control, where 100% represents no change from control and indicated as a horizontal dashed line in graphs. Values above this line indicate an increase in protein carbonyl concentration, and thus a status of more stressed or damaged cell culture. In the single-compound experiments, only one compound was significantly different in a one-sample *t*-test of each % of control value when compared back to a value of 100; DEET had a lower than control amount of protein carbonyl (Figure 4A, *t* = 6.42, df = 2, * *p* = 0.02), suggesting the possibility of a protective effect. However, this effect was not seen in the DEET + metals exposure experiment, as DEET alone compared to control media was not significant (*t* = 0.09, df = 3, *p* = 0.94). There were no significant differences in any of the metals in conjunction with DEET compared to DEET alone, either (Figure 4B, ANOVA F_(9,30)_ = 0.43, *p* = 0.91). No significant effects of PB in conjunction with metals (Figure 4C, ANOVA F_(9,30)_ = 1.48, *p* = 0.20) or of PM in conjunction with metals (Figure 4D, ANOVA F_(9,30)_ = 1.81, *p* = 0.11) were detected either.

### 3.5. Oxidative Stress Proteins

We examined the effects of organic and metal compound exposure in BMEC cell cultures on expression oxidative stress markers (GPX4, catalase, SOD, MMP-3, and MMP-9). In each experimental set, BMECs were cultured alone and exposed to a media control, an organic compound alone, or the organic compound in combination with one of the metals. Graphs are presented as arbitrary units, where the chemiluminescence expression level of the target protein is normalized to the expression level of β-actin within each sample.

**Figure 5:**
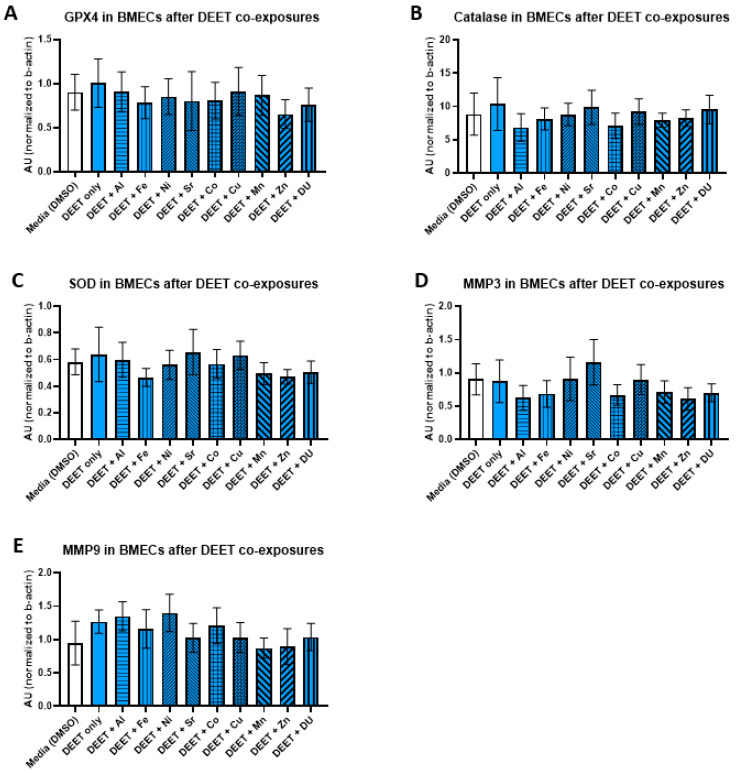
Oxidative stress proteins in brain microvascular endothelial cell (BMEC) cultures after DEET and metal exposure. Assessment of several oxidative stress related protein levels in BMEC cultures after exposure to DEET (10 µM) and metals (1 µM). (**A**) GPX4 expression, (**B**) catalase expression, (**C**) SOD expression, (**D**) MMP3 expression, and (**E**) MMP9 expression. All assessments are from the same experiment setup—each protein run is completed from the same homogenized sample cell pellets. DEET alone is compared to media alone, and then each DEET + metal group is compared to DEET alone. Data represent the mean and standard deviation of three independent experiments.

For the DEET + metals experimental set, there were no significant differences in expression of any protein after any treatment. All protein results are shown in Figure 5 (A) GPX4, F_(10,33)_ = 0.17, *p* = 1.00; (B) catalase, F_(10,33)_ = 0.24, *p* = 0.99); (C) SOD, F_(10,33)_ = 0.34, *p* = 0.96; (D) MMP-3, F_(10,31)_ = 0.48, *p* = 0.89; (E) MMP-9, F_(10,32)_ = 0.54, *p* = 0.81).

**Figure 6:**
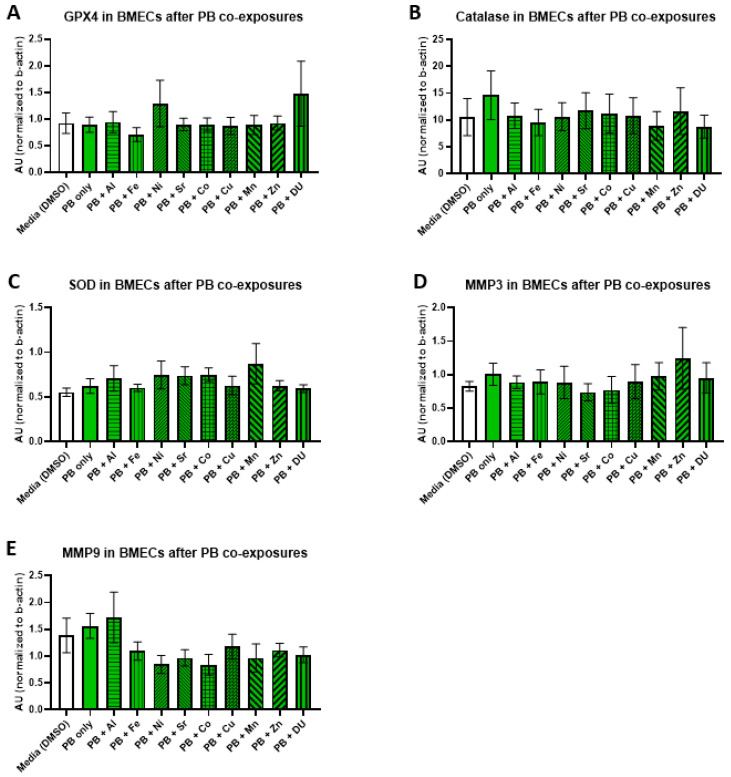
Oxidative stress proteins in brain microvascular endothelial cell (BMEC) cultures after pyridostigmine bromide (PB) and metal exposure. Assessment of several oxidative stress related protein levels in BMEC cultures after exposure to PB (10 µM) and metals (1 µM). (**A**) GPX4 expression, (**B**) catalase expression, (**C**) SOD expression, (**D**) MMP3 expression, and (**E**) MMP9 expression. All assessments are from the same experiment setup—each protein run is completed from the same homogenized sample cell pellets. PB alone is compared to media alone, and then each PB + metal group is compared to PB alone. Data represent the mean and standard deviation of three independent experiments.

For the PB + metals experimental set, there were no significant differences in expression of any protein after any treatment. All protein results are shown in Figure 6 (A) GPX4, F_(10,33)_ = 0.65, *p* = 0.76; (B) catalase, F_(10,33)_ = 0.24, *p* = 0.99); (C) SOD, F_(10,33)_ = 0.70, *p* = 0.72; (D) MMP-3, F_(10,32)_ = 0.36, *p* = 0.95; (E) MMP-9, F_(10,32)_ = 1.34, *p* = 0.25).

**Figure 7:**
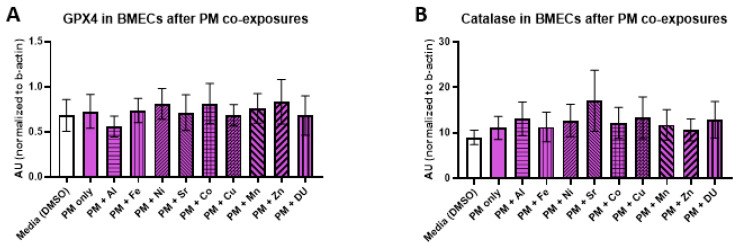

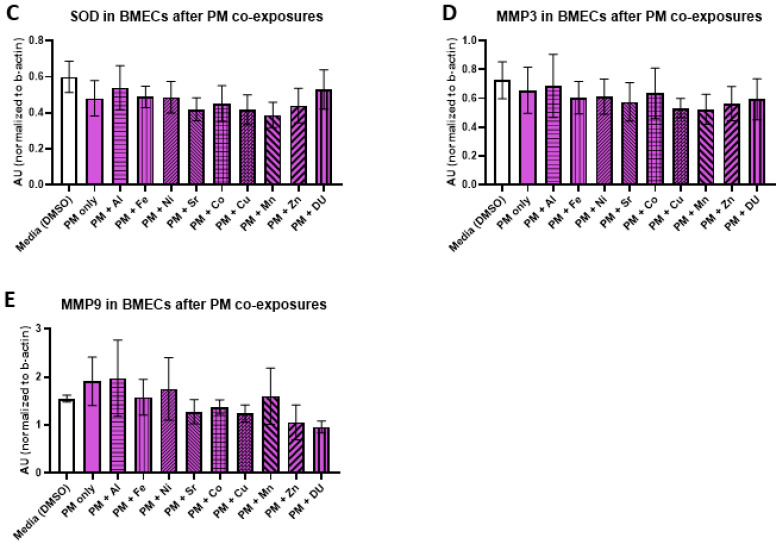
Oxidative stress proteins in brain microvascular endothelial cell (BMEC) cultures after permethrin (PM) and metal exposure. Assessment of several oxidative stress related protein levels in BMEC cultures after exposure to PM (10 µM) and metals (1 µM). (**A**) GPX4 expression, (**B**) catalase expression, (**C**) SOD expression, (**D**) MMP3 expression, and (**E**) MMP9 expression. All assessments are from the same experiment setup—each protein run is completed from the same homogenized sample cell pellets. PM alone is compared to media alone, and then each PM + metal group is compared to PM alone. Data represent the mean and standard deviation of three independent experiments.

For the PM + metals experimental set, there were also no significant differences in expression of any protein after any treatment. All protein results are shown in Figure 7 (A) GPX4, F_(10,32)_ = 0.16, *p* = 1.0; (B) catalase, F_(10,33)_ = 0.29, *p* = 0.98); (C) SOD, F_(10,32)_ = 0.48, *p* = 0.89; (D) MMP-3, F_(10,33)_ = 0.20, *p* = 0.99; (E) MMP-9, F_(10,32)_ = 0.57, *p* = 0.83).

## 4. Discussion

Given the multiple symptom profile associated with Gulf War Illness and that so many military personnel experiencing GWI do not all share known single-exposure scenarios, it is likely that multiple factors are at work. One proposed multi-exposure scenario is a combination of exposure to organic compounds in the field at the time, such as N,N-diethyl-*m*-toluamide (DEET), pyridostigmine bromide (PB), or permethrin (PM), and metals found in the “desert dust” that was inhaled and internalized. The blood–brain barrier serves to heavily restrict movement of molecules between the blood and central nervous system (CNS) [50,51,52]. Most of the properties of the BBB are due to endothelial cells and supporting astrocyte cells, and if this were to be disrupted and allow the passage of compounds such as metals or viral particles into the CNS, it could contribute to the fatigue, anxiety, depression, and other cognitive problems seen in GWI. We hypothesized that exposures of sub-toxic levels of DEET, PB, and PM, particularly in combination with concurrent exposure to sub-toxic levels of metals, would adversely affect the health and viability of cells involved in the BBB, which could lead to subtle changes in permeability and function. In running a dose range of organic compounds on BMECs and astrocytes, we found that in general there was a tipping point between 10 μM and 100 μM in toxicity of both cell types, although this change was much stronger in PM exposure than DEET or PB (Supplementary Materials Figure S2). Similarly, many metal treatments had a tipping point after the 10 μM dose in both BMEC and astrocyte cultures (Supplementary Materials Figures S3 and S4), but enough started a downward trend after only 1 μM that we chose that as an across-the-board sub-toxic dose for subsequent experiments. It is important to note that metals had rather different dose responses, though, and more specific follow-up at other exposure concentrations would be worth examining. For example, Sr stayed close to control toxicity levels in both BMECs and astrocytes across all doses tested, Al actually increased viability markers in BMECs over several doses, and Mn and Cu had downward trending curves from a much lower dose in BMECs and astrocytes. We also report that in BMEC cultures, Co and Mn in conjunction with each of the three organic compounds were significantly more toxic than the corresponding organic compound alone. Mn neurotoxicity has been well studied [53,54,55]. In fact, using RBE cells, a rat brain endothelial cell line, dos Santos and colleagues showed that Mn demonstrated significant toxicity in a dose range of 200–800 µM and concluded that Mn induced mitochondrial injury in these cells [56]. We observed Mn toxicity at much lower concentrations, both alone as well as in combination with Gulf War-associated organics. Although beyond the scope of this study, the role of mitochondria in the potentiation of combined organic/metal exposures, especially in light of the important role the mitochondria play in the production of reactive oxygen and nitrogen species [57], would add to our understanding of these unique exposure scenarios. 

In evaluating oxidative stress markers, DU appears to have the most effect on BMEC cultures. DU alone increased nitric oxide levels, and DEET plus DU exposure increased nitric oxide levels, although DEET alone had no effect. DU increased nitric oxide levels in co-exposure with both the other organic compounds, PB and PM, as well, suggesting internalized DU could be a greater risk to brain function if the person is also exposed to prophylactic or insecticide compounds at the same time. It is not clear whether the increase in nitric oxide in the co-exposure scenarios was the result of a synergistic effect of DU with the organic compound, or solely ascribed to DU. Interestingly, combinations of Sr with each of the three organic compounds reduced nitric oxide production, suggesting a protective effect; this could be tied to the relative lack of toxicity seen across the tested dose range of Sr in cell culture. Strontium has the ability to replace calcium in many systems [58]; however, it is not known whether this property can explain this finding. DEET may also have a propensity to increase protein carbonyl levels in BMECs, but this exposure dose could be on a cusp of toxicity given a significant difference was found in one experiment but not another. We did not see effects of the organic compounds or metals, alone or in combination, on TBARS levels, or oxidative stress marker protein expression of GPX4, catalase, SOD, MMP-3, or MMP-9. Although we did not observe significant effects on the cell’s antioxidant defenses (GPX4, catalase, SOD) or induction of damage-associated proteins (MMP-3, MMP-9) at the doses we tested, there have been reports in the literature suggesting that metal exposure can affect these enzyme systems [59,60,61]. Many of the metals tested can also function as catalysts in the Fenton and Haber-Weiss reactions and are capable of generating highly reactive oxygen and nitrogen species [62,63,64]. It has been postulated that these reactions play a major role in metal-induced neurodegeneration [65,66]. 

A limitation of this study, in fact of any study using an in vitro or animal model system, is the extrapolation to the human condition. In this regard, as noted earlier, record keeping with respect to the various types of exposure, especially pyridostigmine bromide administration, in the First Gulf War were spotty as best. Thus actual in-theatre concentration levels of the various compounds encountered are not known. Experimentally, this necessitates an initial cytotoxicity assessment of the various test compounds being studied. Whether the concentrations used in these in vitro studies correlate to actual human exposure levels in the First Gulf War remain to be seen. 

The effects we report here are subtle, but suggest implications on degraded function of the BBB as a result of these unique Gulf War-related exposures. In future work we will examine changes in BBB function more closely using a BMEC and astrocyte co-culture transwell system as a more accurate 3D model of BBB vasculature, rather than traditional cell culture plates for toxicity assays.

## 5. Conclusions

Human BMEC and human astrocyte cell cultures were evaluated for toxicity across a range of concentrations of organic compounds (N,N-diethyl-*m*-toluamide (DEET), permethrin, and pyridostigmine bromide) and metals (aluminum, iron, nickel, strontium, cobalt, copper, manganese, zinc, and depleted uranium) that are relevant to potential exposure scenarios for US military personnel during the Gulf War. We determined a general sub-toxic dose of 10 µM for the three organic compounds and 1 µM for the nine metals of interest and used this to examine markers of viability and oxidative stress on BMEC cell cultures in co-exposure scenarios for the organic and metal compounds, as these would be more relevant to actual exposure scenarios for US military personnel and thus may contribute to Gulf War Illness symptoms. Many markers were not affected, or were subtle, but we found a significant increase in nitric oxide after exposure to any of the three compounds in conjunction with depleted uranium. Future work will determine how much, if any, effect this has on blood–brain barrier function.

## Supplementary Materials

The following are available online at https://www.mdpi.com/1660-4601/17/22/8336/s1, Figure S1: Immunohistochemistry of BMEC markers; Figure S2: Toxicity of organic compounds on BMECs and astrocytes; Figure S3: Toxicity of metals on BMECs; Figure S4: Toxicity of metals on astrocytes; Figure S5a: Microscopy of BMECs exposed to a dose range of organic or metal compounds; Figure S5b: Microscopy of BMECs exposed to a dose range of organic or metal compounds; Figure S6a: Microscopy of astrocytes exposed to a dose range of organic or metal compounds; Figure S6b: Microscopy of astrocytes exposed to a dose range of organic or metal compounds; Figure S7: Example of ProteinSimple run data; Table S1: Antibody Table.
