# Supplementary material for: Effects of Incubation of Human Brain Microvascular Endothelial Cells and Astrocytes with Pyridostigmine Bromide, DEET, or Permethrin in the Absence or Presence of Metal Salts
Source: Int J Environ Res Public Health. 2020 Nov 11;17(22):8336. doi: 10.3390/ijerph17228336 (PMC7696739; doi:10.3390/ijerph17228336)
Supplement: Supplementary file 1 [file ijerph-17-08336-s001.pdf]

Figure Legends for Supplementary Information (IJERPH-969865 Hoffman_Kalinich)

Table S1 –Antibody Table
Details for antibodies used in fluorescent immunohistochemistry and protein expression analysis.

Figure S1 – Immunohistochemistry of BMEC markers
Microscopy images of BMEC cultures to verify expression of BBB relevant markers occludin and ZO-1 using fluorescent immunohistochemistry. Images are at 10X magnification, scale bar represents 50 µm. A) image of BMEC culture stained with occludin in red, DAPI (for nuclei) in blue. B) image of BMEC culture stained with ZO-1 in red, DAPI in blue.

Figure S2 – Toxicity of organic compounds on BMECs and astrocytes
Dose response curves of individual treatments with organic compounds in BMEC (A-F) or astrocyte (G-L) cultures. Toxicity and viability were determined with MTT or NR assays for each cell type and organic combination. Dotted line at 100 indicates 100% of control measurement: values below the line indicate the dose is toxic to the cells and viability is decreased. Data represent the mean and standard deviation of three independent experiments.

Figure S3 – Toxicity of metals on BMECs
Dose response curves of individual treatments with metals in BMEC cultures. Toxicity and viability were determined with MTT (A-I) or NR (J-R) assays for each cell type and organic combination. Dotted line at 100 indicates 100% of control measurement: values below the line indicate the dose is toxic to the cells and viability is decreased. Data represent the mean and standard deviation of three independent experiments.

Figure S4 – Toxicity of metals on astrocytes
Dose response curves of individual treatments with metals in astrocyte cultures. Toxicity and viability were determined with MTT (A-I) or NR (J-R) assays for each cell type and organic combination. Dotted line at 100 indicates 100% of control measurement: values below the line indicate the dose is toxic to the cells and viability is decreased. Data represent the mean and standard deviation of three independent experiments.

Figure S5a – Microscopy of BMECs exposed to a dose range of organic or metal compounds
Giemsa stained BMEC cell cultures after exposure to metals or organic compounds, paired with Supplemental Figure 4b.

Figure S5b – Microscopy of BMECs exposed to a dose range of organic or metal compounds
Giemsa stained BMEC cell cultures after exposure to metals or organic compounds, paired with Supplemental Figure 4a.

Figure S6a – Microscopy of astrocytes exposed to a dose range of organic or metal compounds
Giemsa stained astrocyte cell cultures after exposure to metals or organic compounds, paired with Supplemental Figure 5b.

Figure S6b – Microscopy of astrocytes exposed to a dose range of organic or metal compounds
Giemsa stained astrocyte cell cultures after exposure to metals or organic compounds, paired with Supplemental Figure 5a.

Figure S7 – Example of ProteinSimple run data

Rather than a traditional Western blot with gels and membranes for analysis of protein expression, the ProteinSimple Wes system uses a capillary-based size separation method and chemiluminescent detection. Analysis of band intensity is performed directly in the Wes machine, automatically calculating area under the peak. The Compass program software is also capable of generating an image like that of a traditional Western blot. The chemiluminescence values for a sample set of DEET+metal BMEC experiment is shown in (A), and the corresponding “Western blot” image generated from those values is shown in (B). GPX4 and β-actin are labeled in both images.

Antibody Table

Primary Antibodies					
**Antibody**	**Host**	**Vendor, Cat #**	**Purpose**	**Conc.**	**Size (kDa)**
ZO-1	Rb	ThermoFisher 40-2200	BBB marker	1:50	
Occludin (N-term)	Rb	ThermoFisher 40-6100	BBB marker	1:50	
Alexa Fluor 555 (red)	Dnk	Abcam ab150074	Fluorescent 2°	1:1000	
DAPI (blue)		ThermoFisher D1306	Nuclei stain	1 µg/ml	
Superoxide dismutase (SOD)	Rb	Abcam, Ab13498	Antioxidant defense	1:50	25
Glutathione peroxidase (GPX4)	Ms	Novusbio, MAB5457	Antioxidant defense	1:50	25
Catalase	Ms	Novusbio, MAB3398	Antioxidant defense	1:200	62
MMP3	Rb	Abcam, Ab53015	Cell damage	1:50	54
MMP9	Rb	Abcam, 38898	Cell damage	1:50	92
Beta-actin (13E5) x rab		CellSignaling, 4970S	Housekeeping (normalization)	1:10,000	48
Beta-actin (8H10D10) x mou		CellSignaling, 3700S	Housekeeping (normalization)	1:10,000	48

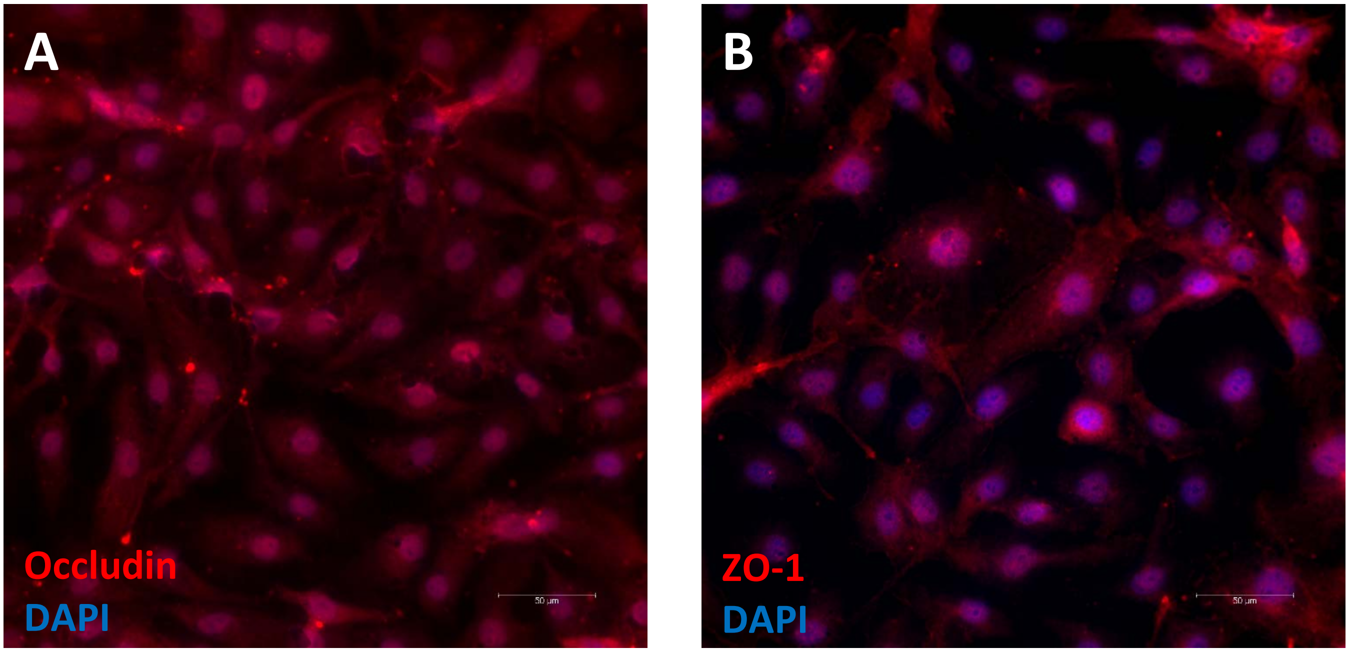
A
Occludin
DAPI
50 µm
B
ZO-1
DAPI
50 µm

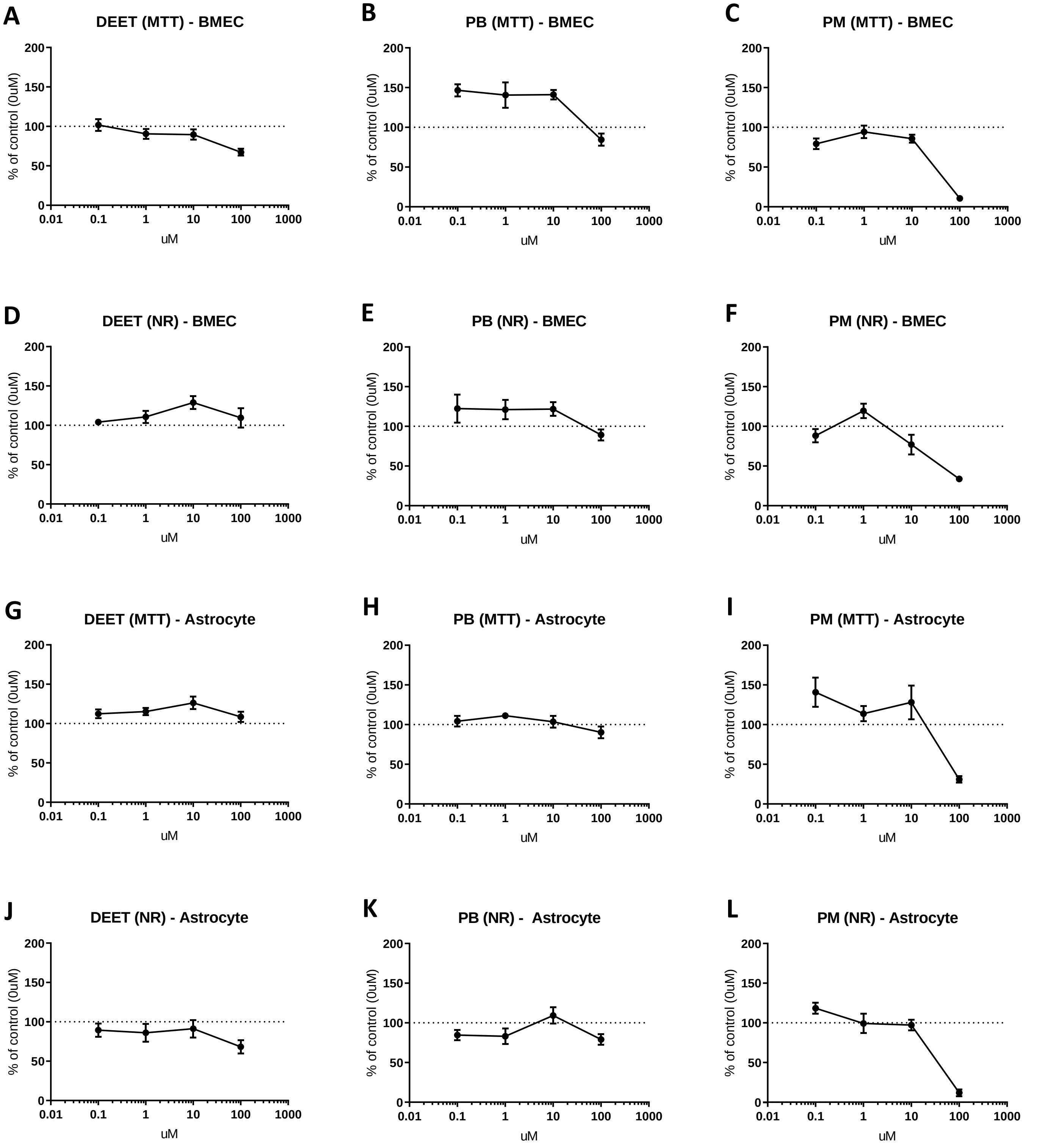
A
DEET (MTT) - BMEC
B
PB (MTT) - BMEC
C
PM (MTT) - BMEC
D
DEET (NR) - BMEC
E
PB (NR) - BMEC
F
PM (NR) - BMEC
G
DEET (MTT) - Astrocyte
H
PB (MTT) - Astrocyte
I
PM (MTT) - Astrocyte
J
DEET (NR) - Astrocyte
K
PB (NR) - Astrocyte
L
PM (NR) - Astrocyte
% of control (0uM)
0
50
100
150
200
uM
0.01
0.1
1
10
100
1000

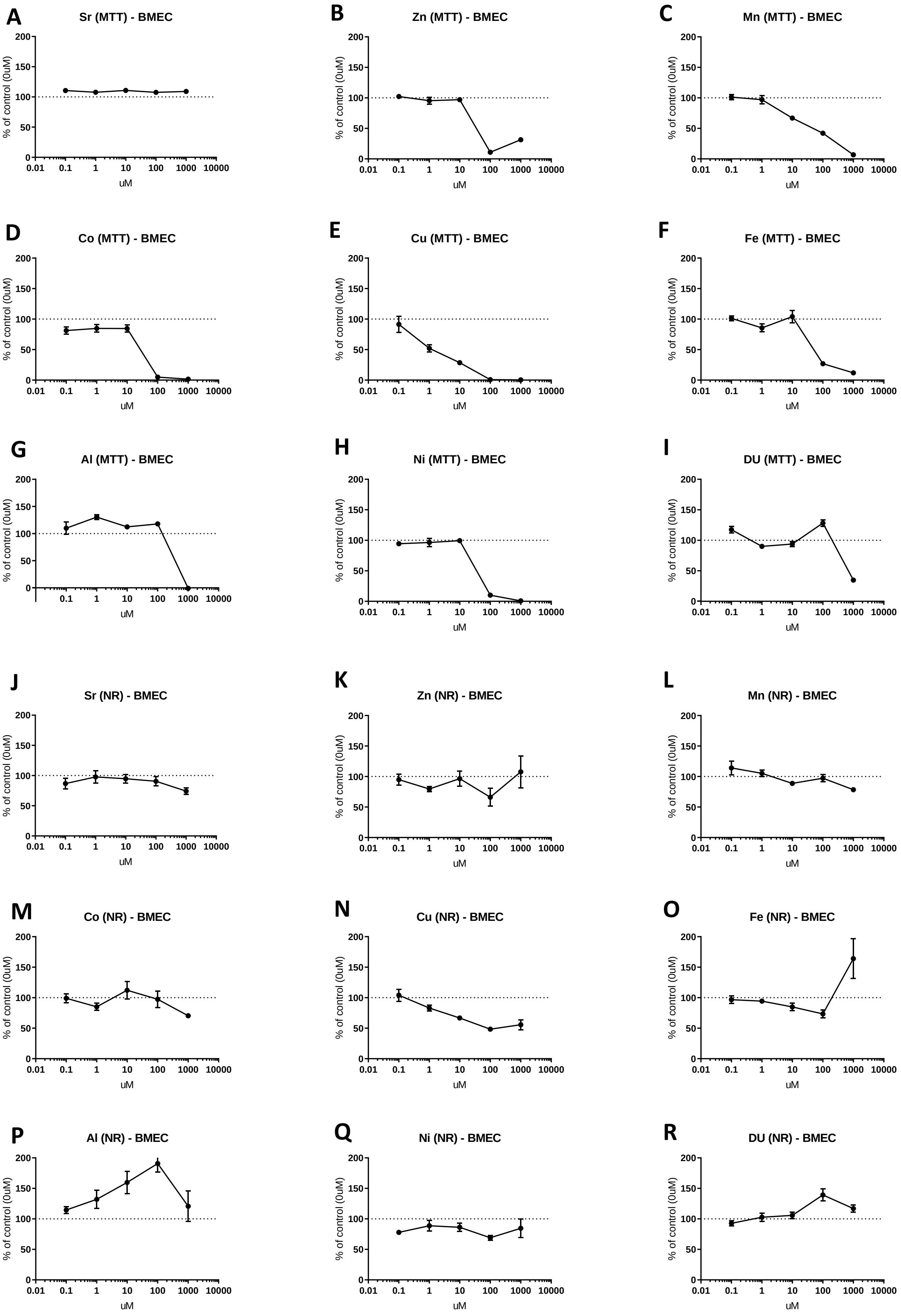

A
Sr (MTT) - BMEC
B
Zn (MTT) - BMEC
C
Mn (MTT) - BMEC
D
Co (MTT) - BMEC
E
Cu (MTT) - BMEC
F
Fe (MTT) - BMEC
G
Al (MTT) - BMEC
H
Ni (MTT) - BMEC
I
DU (MTT) - BMEC
J
Sr (NR) - BMEC
K
Zn (NR) - BMEC
L
Mn (NR) - BMEC
M
Co (NR) - BMEC
N
Cu (NR) - BMEC
O
Fe (NR) - BMEC
P
Al (NR) - BMEC
Q
Ni (NR) - BMEC
R
DU (NR) - BMEC
% of control (0uM)
0
50
100
150
200
uM
0.01
0.1
1
10
100
1000
10000

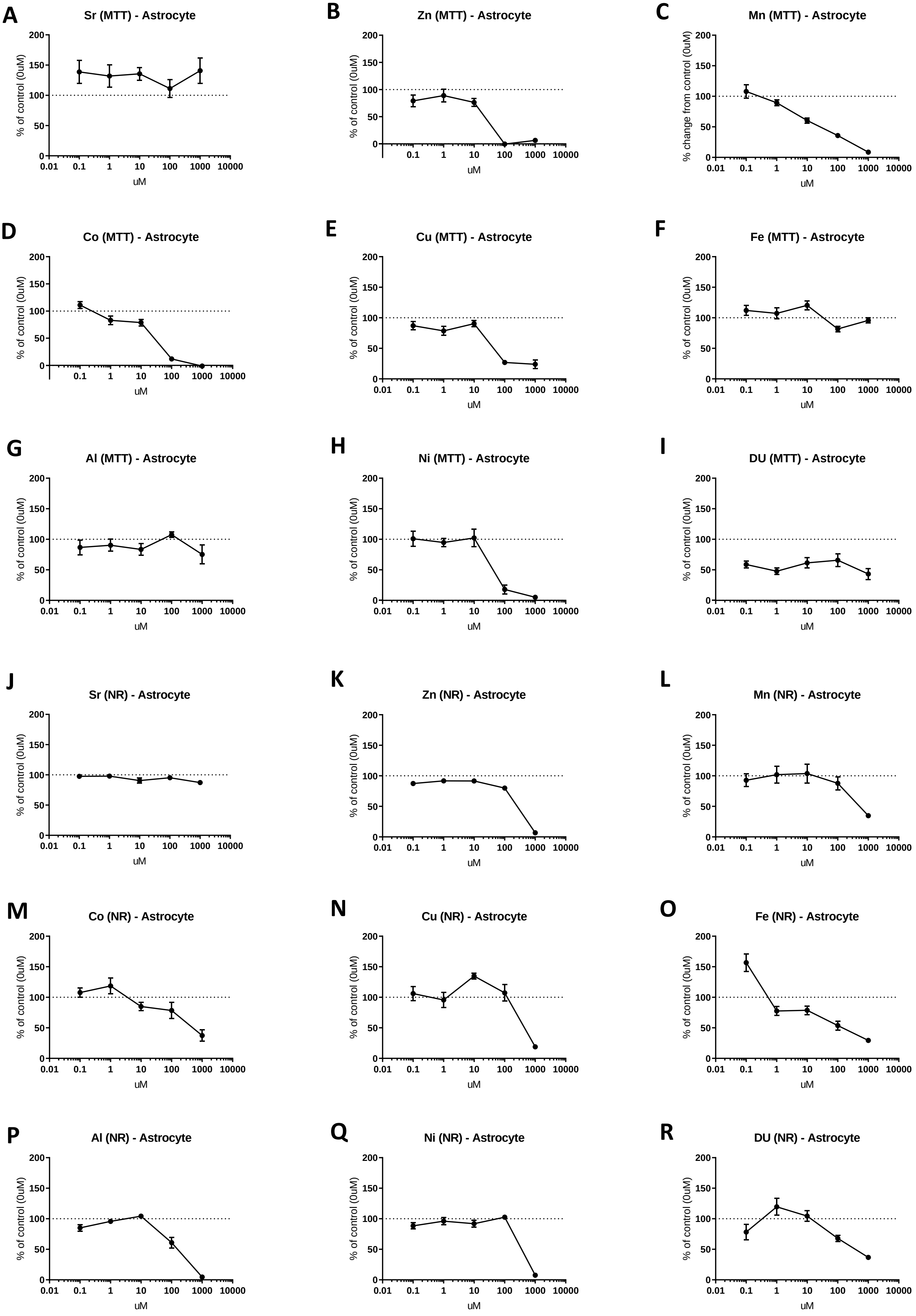

A
Sr (MTT) - Astrocyte
% of control (0uM)
uM
B
Zn (MTT) - Astrocyte
% of control (0uM)
uM
C
Mn (MTT) - Astrocyte
% change from control (0uM)
uM
D
Co (MTT) - Astrocyte
% of control (0uM)
uM
E
Cu (MTT) - Astrocyte
% of control (0uM)
uM
F
Fe (MTT) - Astrocyte
% of control (0uM)
uM
G
Al (MTT) - Astrocyte
% of control (0uM)
uM
H
Ni (MTT) - Astrocyte
% of control (0uM)
uM
I
DU (MTT) - Astrocyte
% of control (0uM)
uM
J
Sr (NR) - Astrocyte
% of control (0uM)
uM
K
Zn (NR) - Astrocyte
% of control (0uM)
uM
L
Mn (NR) - Astrocyte
% of control (0uM)
uM
M
Co (NR) - Astrocyte
% of control (0uM)
uM
N
Cu (NR) - Astrocyte
% of control (0uM)
uM
O
Fe (NR) - Astrocyte
% of control (0uM)
uM
P
Al (NR) - Astrocyte
% of control (0uM)
uM
Q
Ni (NR) - Astrocyte
% of control (0uM)
uM
R
DU (NR) - Astrocyte
% of control (0uM)
uM
0.01 0.1 1 10 100 1000 10000
0 50 100 150 200

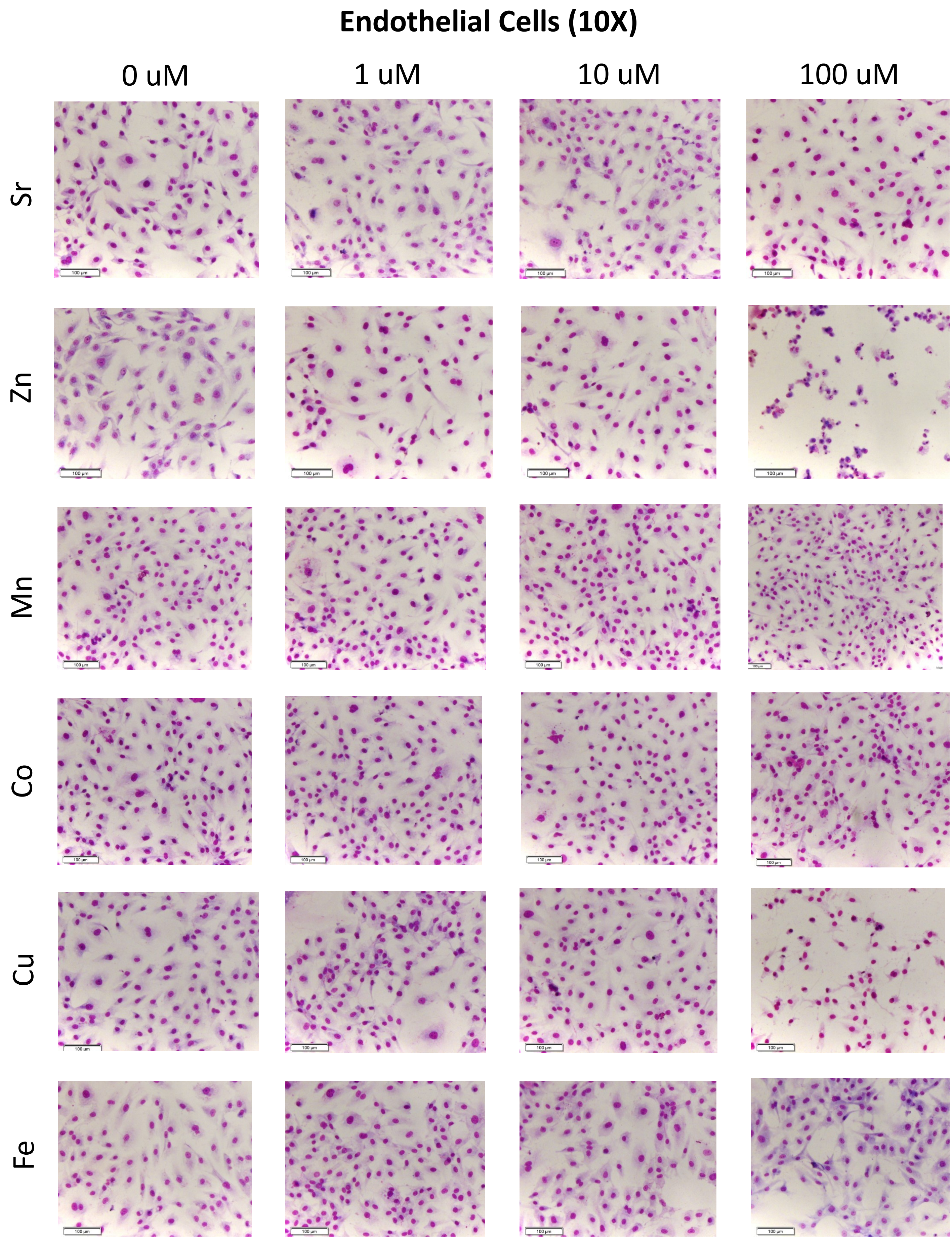
Endothelial Cells (10X)
0 uM
1 uM
10 uM
100 uM
Sr
Zn
Mn
Co
Cu
Fe

Endothelial Cells (10X)

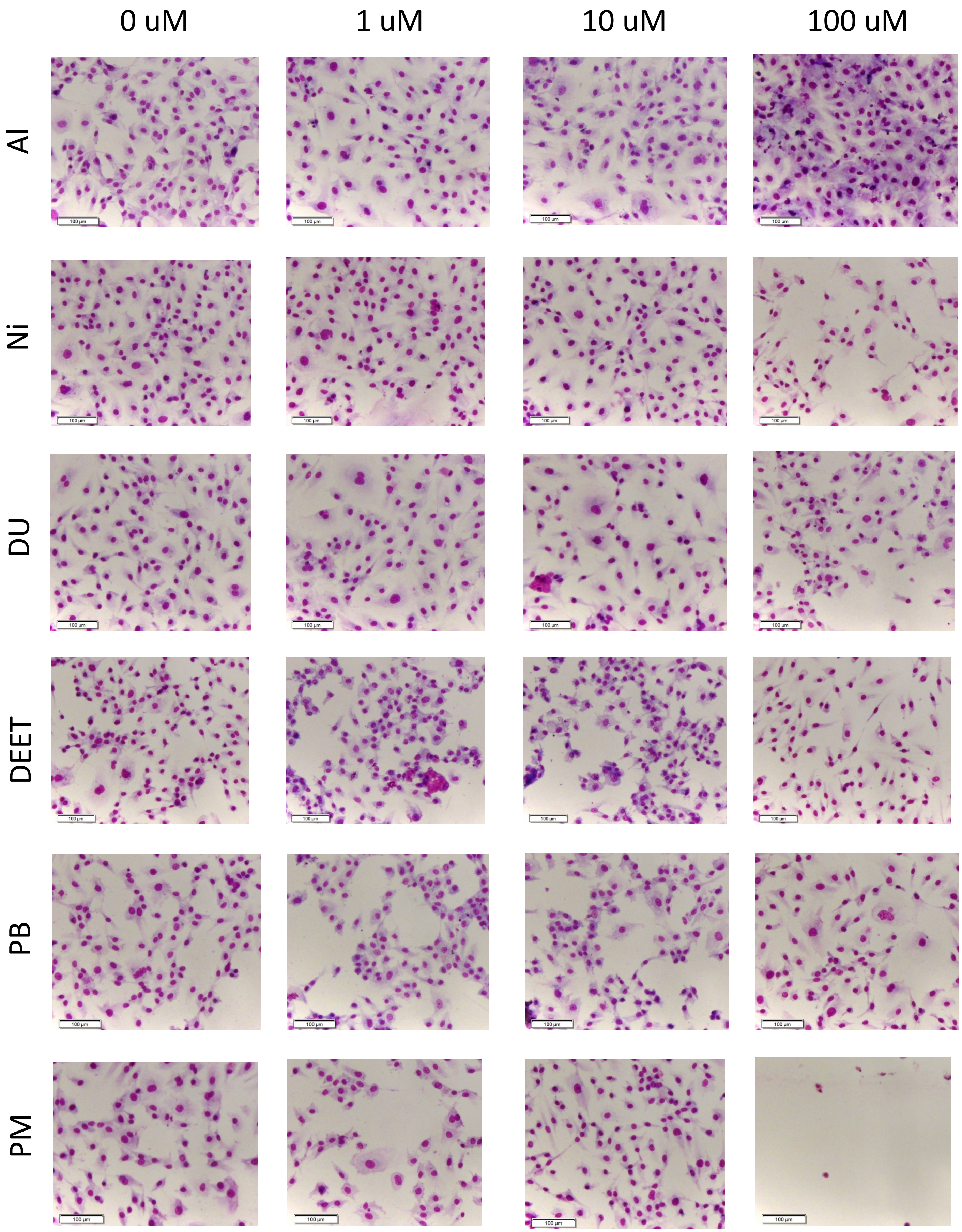

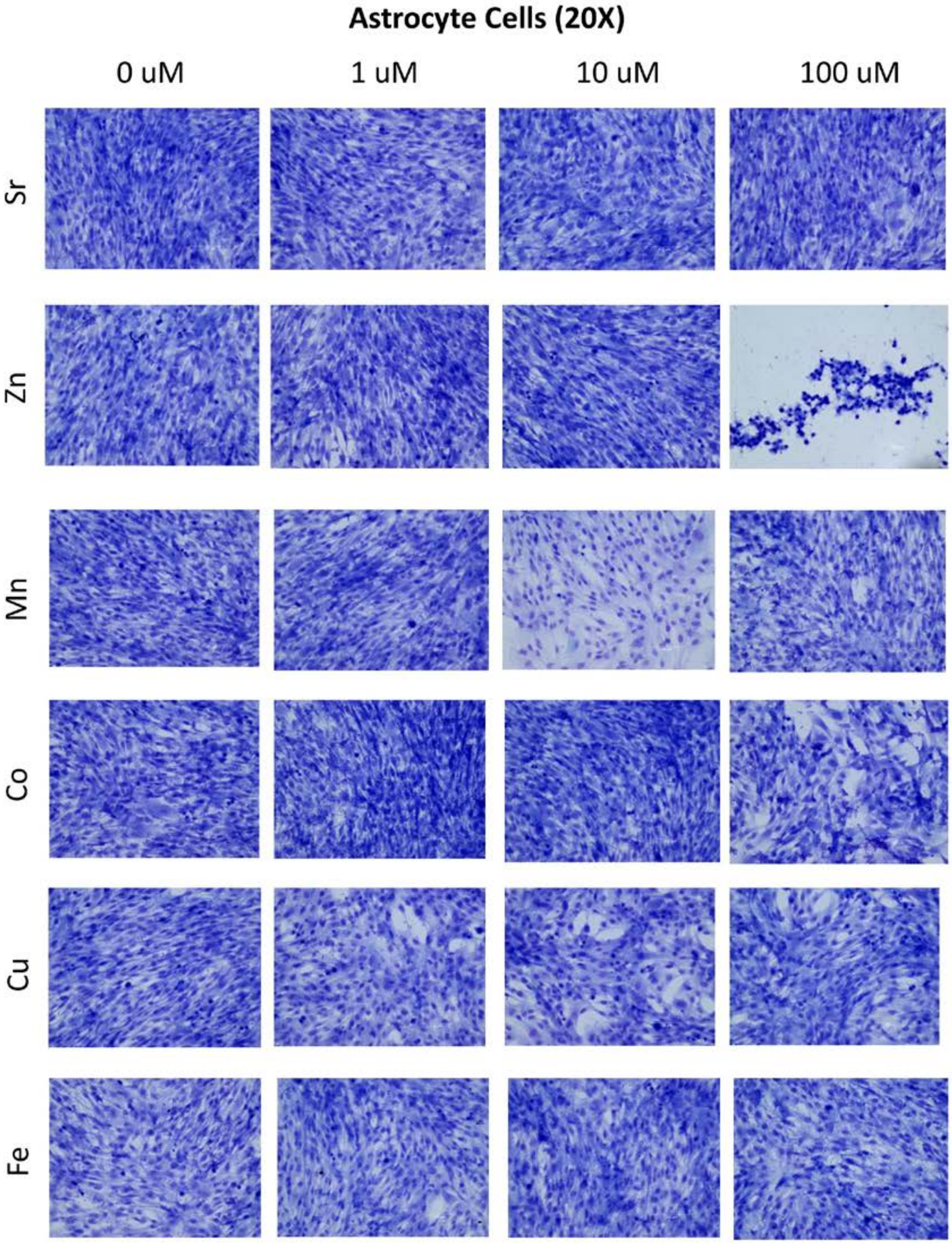
Astrocyte Cells (20X)
0 uM
1 uM
10 uM
100 uM
Sr
Zn
Mn
Co
Cu
Fe

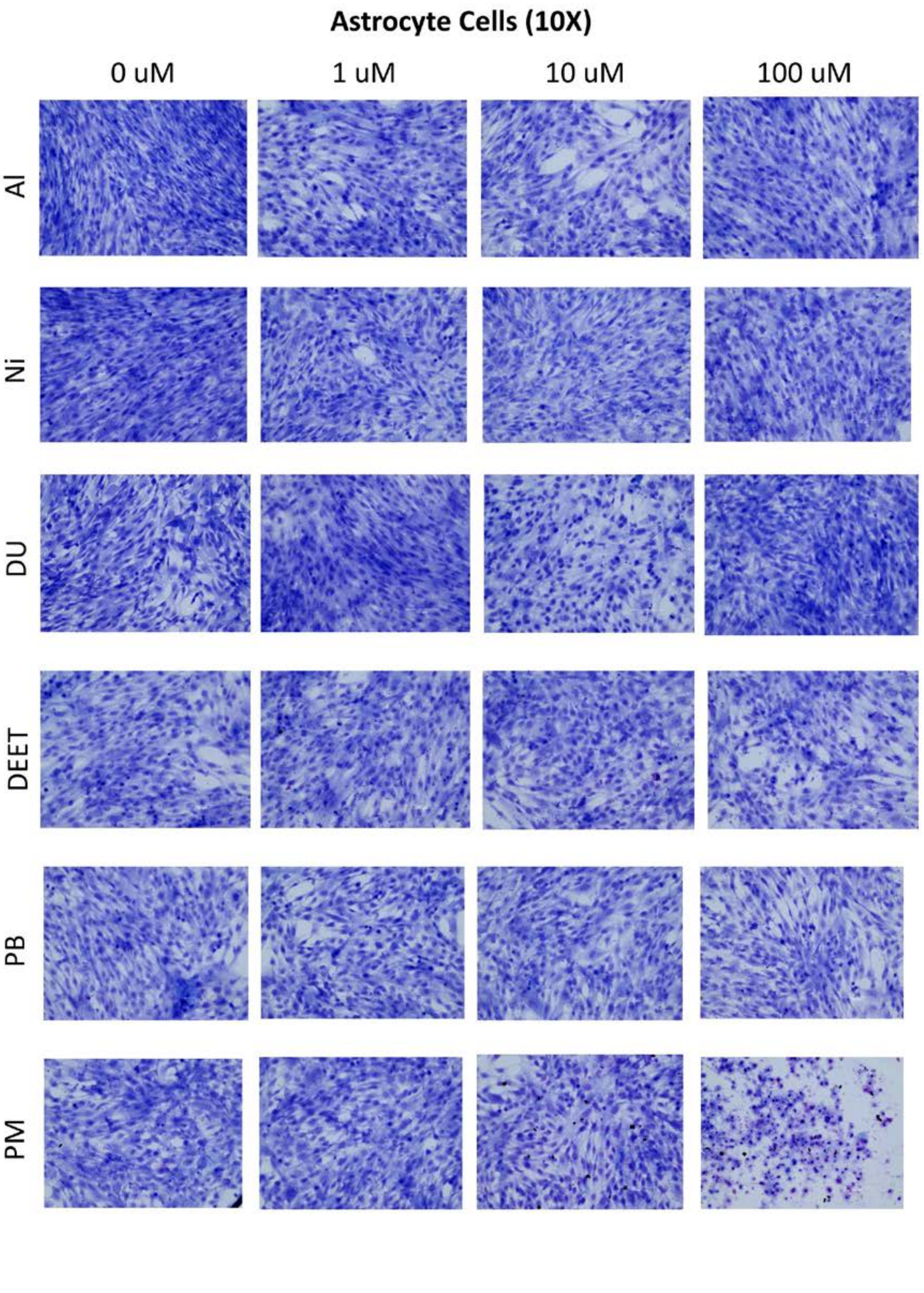
Astrocyte Cells (10X)
0 uM
1 uM
10 uM
100 uM
Al
Ni
DU
DEET
PB
PM

A

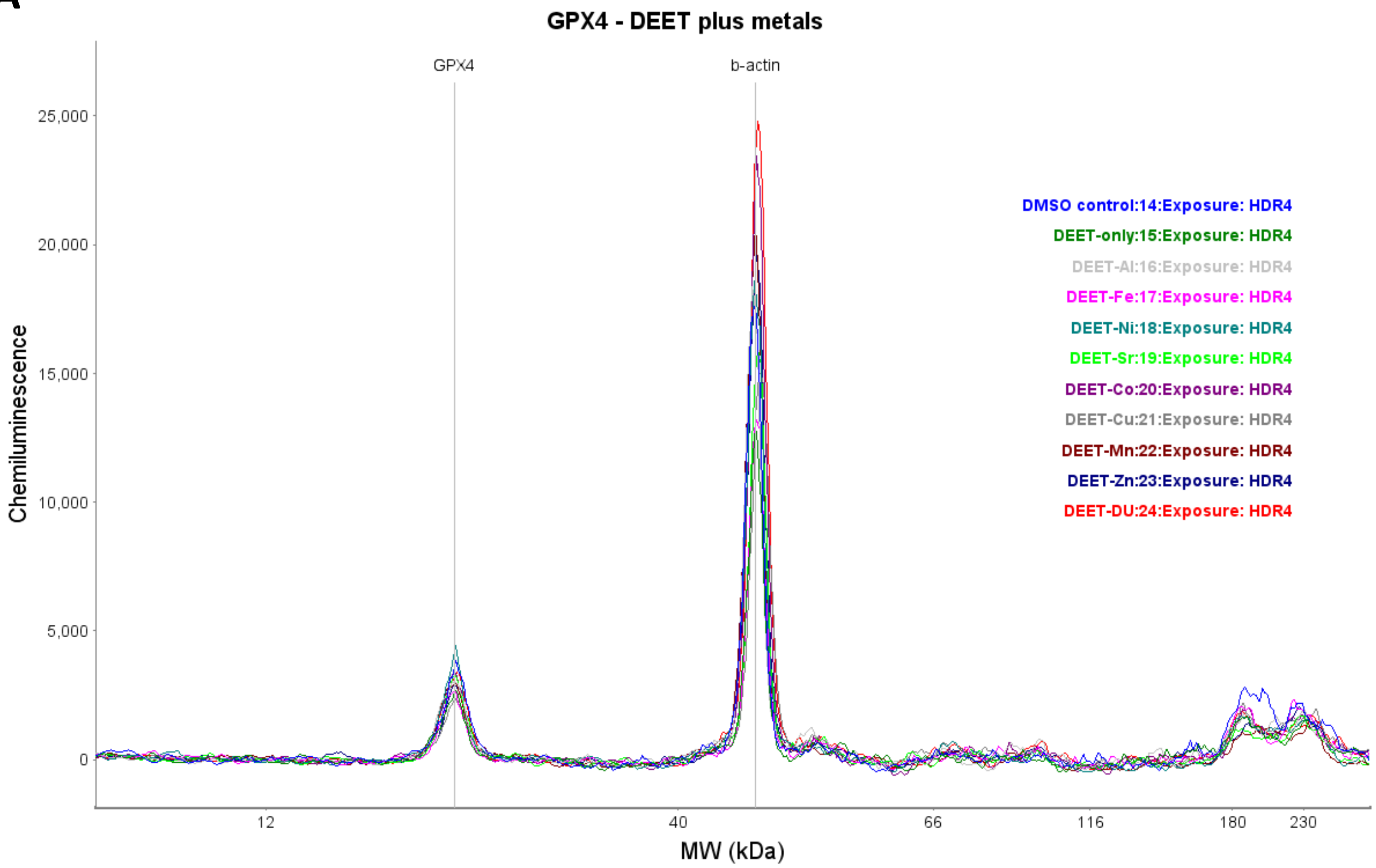
GPX4 - DEET plus metals
GPX4
b-actin
Chemiluminescence
25,000
20,000
15,000
10,000
5,000
0
12
40
66
116
180
230
MW (kDa)
DMSO control:14:Exposure: HDR4
DEET-only:15:Exposure: HDR4
DEET-Al:16:Exposure: HDR4
DEET-Fe:17:Exposure: HDR4
DEET-Ni:18:Exposure: HDR4
DEET-Sr:19:Exposure: HDR4
DEET-Co:20:Exposure: HDR4
DEET-Cu:21:Exposure: HDR4
DEET-Mn:22:Exposure: HDR4
DEET-Zn:23:Exposure: HDR4
DEET-DU:24:Exposure: HDR4

B

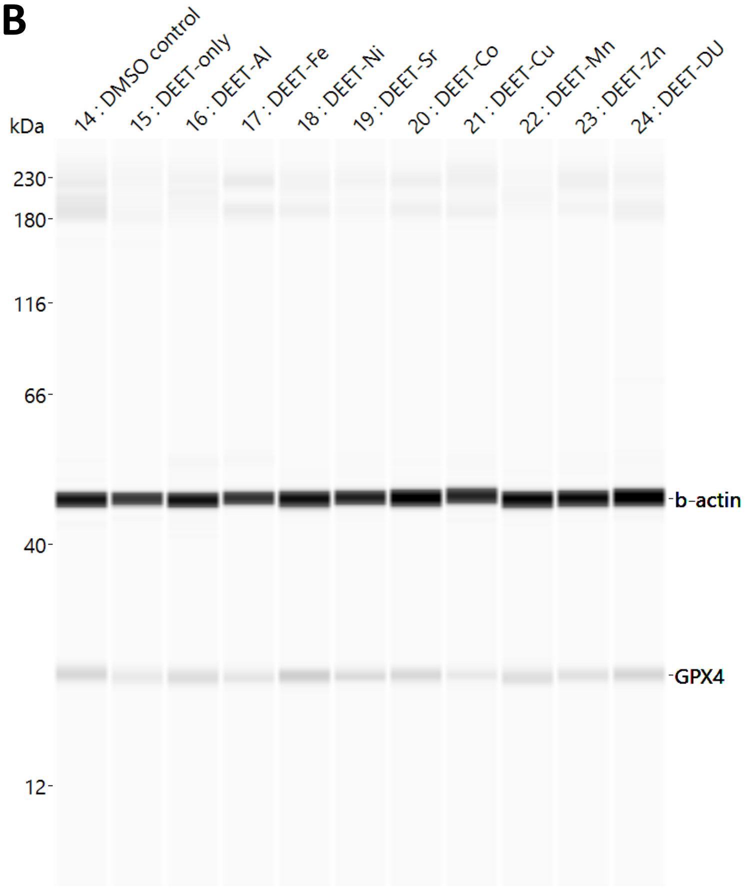
14 : DMSO control
15 : DEET-only
16 : DEET-Al
17 : DEET-Fe
18 : DEET-Ni
19 : DEET-Sr
20 : DEET-Co
21 : DEET-Cu
22 : DEET-Mn
23 : DEET-Zn
24 : DEET-DU
kDa
230
180
116
66
40
12
b-actin
GPX4
